# Quality assessment of lupus nephritis health information on China’s mainstream short-form video platforms: A cross-sectional study

**DOI:** 10.1097/MD.0000000000049924

**Published:** 2026-07-24

**Authors:** Xuemin Yin, Xi Fang, Yinjun Liao

**Affiliations:** a Department of Nephrology, The Second Xiangya Hospital, Central South University, Changsha, China; b Key Laboratory of Kidney Disease and Blood Purification in Hunan Province, Changsha, China; c Department of Anesthesiology, The Second Xiangya Hospital, Central South University, Changsha, China.

**Keywords:** Bilibili, lupus nephritis, short videos, TikTok, video quality

## Abstract

Lupus nephritis (LN) is an important cause of end-stage renal disease. Recently, more and more patients have been accessing LN-related health information through TikTok and Bilibili, but the quality of the content is uneven. This cross-sectional study aims to systematically evaluate the content quality of LN-related short videos on these 2 major platforms. On December 13, 2025, we searched for “LN” in Chinese on TikTok and Bilibili and obtained the top 100 results from each platform. After screening, 159 videos were analyzed. The Global Quality Score (GQS) and modified DISCERN (mDISCERN) scales were used for multidimensional quality evaluation, and the correlation between video characteristics and interaction metrics was analyzed. The results showed that Bilibili’s videos had significantly longer durations (median: 486 seconds vs TikTok’s 82.5 seconds) and higher GQS scores (median: 3.00 vs 2.00, *P* <.05). After adjusting for confounding factors, there was no significant difference in GQS (odds ratio = 1.40, 95% confidence interval: 0.43–4.64) or mDISCERN (odds ratio = 0.78, 95% confidence interval: 0.28–2.16) between TikTok and Bilibili. TikTok videos were more interactive, receiving more likes, shares, and comments. The content on both platforms mainly focused on “treatment” and “symptoms” while rarely covering topics such as “prevention” and “epidemiology.” A positive correlation was found between content quality and the professional background of the creator: the median GQS and mDISCERN scores for videos released by professional clinicians were 3.00, whereas those for individual users were only 1.00. In addition, audience interaction was negatively correlated with content quality. In summary, videos on both Bilibili and TikTok demonstrated modest overall quality.

## 1. Introduction

Systemic lupus erythematosus (SLE) is a multisystem autoimmune disease, and the kidney is one of the most commonly affected target organs.^[1]^ Lupus nephritis (LN) is an important complication of SLE, with an incidence rate of up to 50%, which not only significantly increases morbidity and mortality but also is one of the main causes of progression to chronic kidney disease and end-stage renal disease (ESRD).^[2]^ Studies show that uncontrolled LN can accelerate the occurrence of cardiovascular disease and induce secondary immunodeficiency, exposing patients to a higher risk of infection and cardiovascular events.^[3–5]^ Despite the recent progress in immunosuppressants and biological treatments, 10% to 30% of patients eventually progress to ESRD requiring renal replacement therapy.^[2,6,7]^ In addition, male patients, those with an early onset of the disease, and individuals with comorbidities such as obesity, hypertension, or diabetes experience faster deterioration of renal function and face greater treatment challenges.^[2,8]^ However, in clinical practice, due to the diverse manifestations of the disease, its insidious progression, and the lack of public awareness, many patients are not diagnosed until there is obvious proteinuria or a decline in renal function, thus missing the optimal window for intervention. Therefore, early identification, standardized treatment, and long-term management are crucial for improving the prognosis of patients.

With the development of Internet technology, especially the popularity of mobile social media, more and more patients and their families are turning to online platforms to obtain medical information. This helps with decision-making, relieves anxiety, and improves self-management capabilities.^[9,10]^ In recent years, short-video platforms such as TikTok and Bilibili have risen rapidly. With their characteristics of fragmented communication, intuitive presentation, and strong interactivity, they have become important channels for the public, especially young people, to access health knowledge.^[11,12]^ According to statistics, by 2024, TikTok’s global monthly active users had reached 1.58 billion, and Bilibili’s domestic monthly active users had exceeded 348 million.^[9]^ Although such short videos have significant advantages in terms of communication efficiency and accessibility, their content quality is uneven in different diseases. There may be a risk of encountering inaccurate information, content lacking scientific basis.^[13]^ Research on premature ovarian failure and thyroid eye disease has found that TikTok videos score significantly higher in quality than those on Bilibili.^[14,15]^ However, studies on knee osteoarthritis and prostate cancer have observed opposite or inconsistent results,^[16,17]^ which hints that the quality differences between platforms might be disease-specific. Previous studies have shown that in the context of SLE, the clarity and reliability of Bilibili videos are of better quality than those of TikTok.^[5]^ However, so far, no study has systematically evaluated the quality of content related to LN on mainstream short-video platforms in China, especially TikTok and Bilibili.

This study aims to systematically evaluate LN-related short-video content on TikTok and Bilibili, comparatively reveal the differences in health information quality between the 2 platforms, and provide an exploratory discussion of possible contributing factors to these differences.

## 2. Methods

### 2.1. Data collection

In order to systematically evaluate the information quality of LN on China’s mainstream short-video platforms, this cross-sectional study selected 2 platforms, TikTok and Bilibili, as the objects of analysis. Because the data we collected were only short videos and did not involve any animal or human tissues or specimens, ethical review was not required for this study. To minimize bias caused by personalized recommendation algorithms, a standardized data collection process was adopted. Specifically, on December 13, 2025, a newly registered account with no previous browsing history was used to search for the Chinese keyword “lupus nephritis” in the platform search bars. As most users tend to view only the first few pages of search results, the top 100 videos sorted by relevance were extracted from each platform. Then, 2 researchers independently screened the initial 200 videos. Exclusion criteria included content unrelated to LN, commercial advertisements or medical promotions, repeated uploads, and videos published within the past week. Recently uploaded videos were excluded because they lack complete algorithmic distribution, meaning their engagement metrics (e.g., likes and collections) are still fluctuating rapidly and thus cannot accurately indicate true exposure or popularity. After strict screening, 159 eligible videos were finally included for follow-up analysis (TikTok: n = 98; Bilibili: n = 61; Fig. S1, Supplemental Digital Content 1).

### 2.2. Study groups

According to the identity of the video uploader, all included videos were divided into 4 groups: specialists, nonspecialists, institutions, and individual users. Specialists were doctors or researchers who explicitly identified themselves as nephrologists, with account information indicating verifiable professional qualifications. Nonspecialists referred to practicing physicians who explicitly stated that they worked in clinical departments other than nephrology, with account information disclosing a verified professional medical background. Institutions included official accounts operated by government health departments, hospital public health promotion departments, or medical academic institutions. Individual users included all personal content creators who did not have publicly verifiable medical professional qualifications and were not operating official accounts of any medical or health science institutions.

### 2.3. Quality assessment

Video quality was evaluated using the Global Quality Scale (GQS) and the modified DISCERN tool (mDISCERN). The GQS is a 5-point scale (1 = extremely poor, 5 = excellent) used to subjectively assess overall video quality, structural integrity, and comprehensiveness of information. The mDISCERN scale, adapted from the classic DISCERN tool, is a simplified version containing 5 core questions that focus on assessing the credibility and accuracy of information.^[18–20]^ Two uniformly trained researchers (researcher A and researcher B) independently and blindly scored all 159 videos. When the score difference between the 2 scorers exceeded 15%, a third senior researcher (researcher C) was consulted to arbitrate and make the final assessment.

### 2.4. Statistical analysis

Continuous variables with non-normal distribution were represented by median and interquartile range (IQR), and categorical variables were expressed as frequency and percentage. The Mann–Whitney *U* test was used for comparison between the 2 groups. The Kruskal–Wallis *H* test was used for comparisons involving 3 or more groups. Spearman rank correlation coefficient was employed to analyze the relationship between video characteristics and each quality score. Cohen’s kappa was applied for quantify inter-rater reliability, and ordinal logistic regression was conducted to compare the quality of different platforms. All statistical tests were two-tailed, and *P* <.05 was considered statistically significant.

## 3. Results

### 3.1. Data characteristics

**Figure 1.**
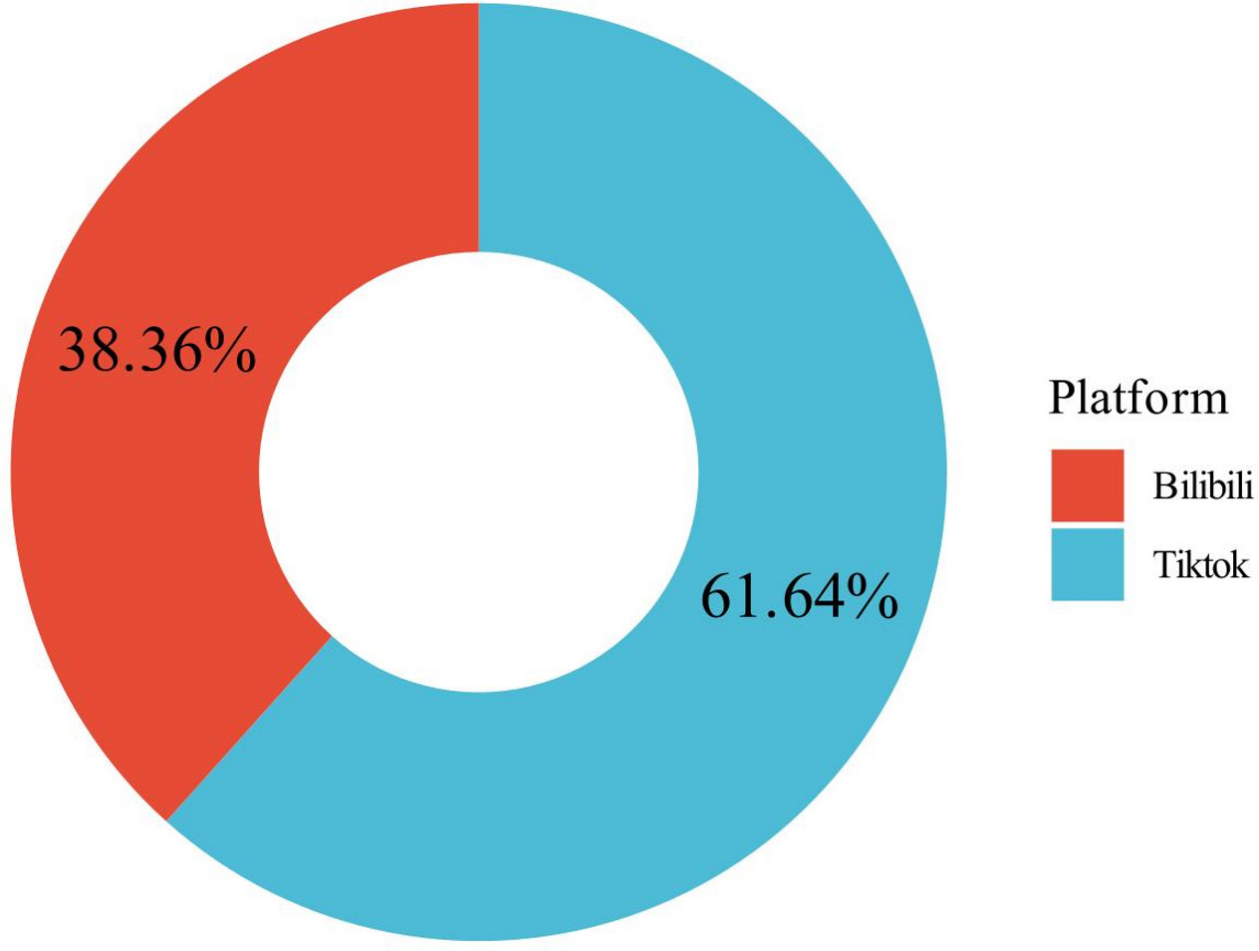
Proportion of lupus nephritis-related videos on Bilibili and TikTok.

The final analysis included 159 videos related to LN (Fig. S1, Supplemental Digital Content 1), of which 98 (61.64%) were from the TikTok platform and 61 (38.36%) were from the Bilibili platform (Fig. 1). The video uploaders included 82 medical professionals, 40 nonmedical professionals, 13 institutions, and 24 individual users. The kappa values of the 2 researchers for GQS and mDISCERN were 0.83 and 0.93, respectively, indicating good consistency. Table S1, Supplemental Digital Content 2, shows that specialists account for the largest proportion (51.57%), and institutions account for the smallest proportion (8.18%). Nonspecialists accounted for 40 (25.16%), and individual users accounted for 24 (15.09%). The median values of likes, collections, comments, shares, and video length across all videos are presented in Table S1, Supplemental Digital Content 2. Among all the videos from the 2 platforms, the total score of topics related to treatment is the highest, reaching 111 points (accounting for 28.24%), while the score for epidemiology is the lowest, at 17 points (accounting for 4.33%; Table S1, Supplemental Digital Content 2).

### 3.2. Uploader characteristics by platform

**Figure 2.**
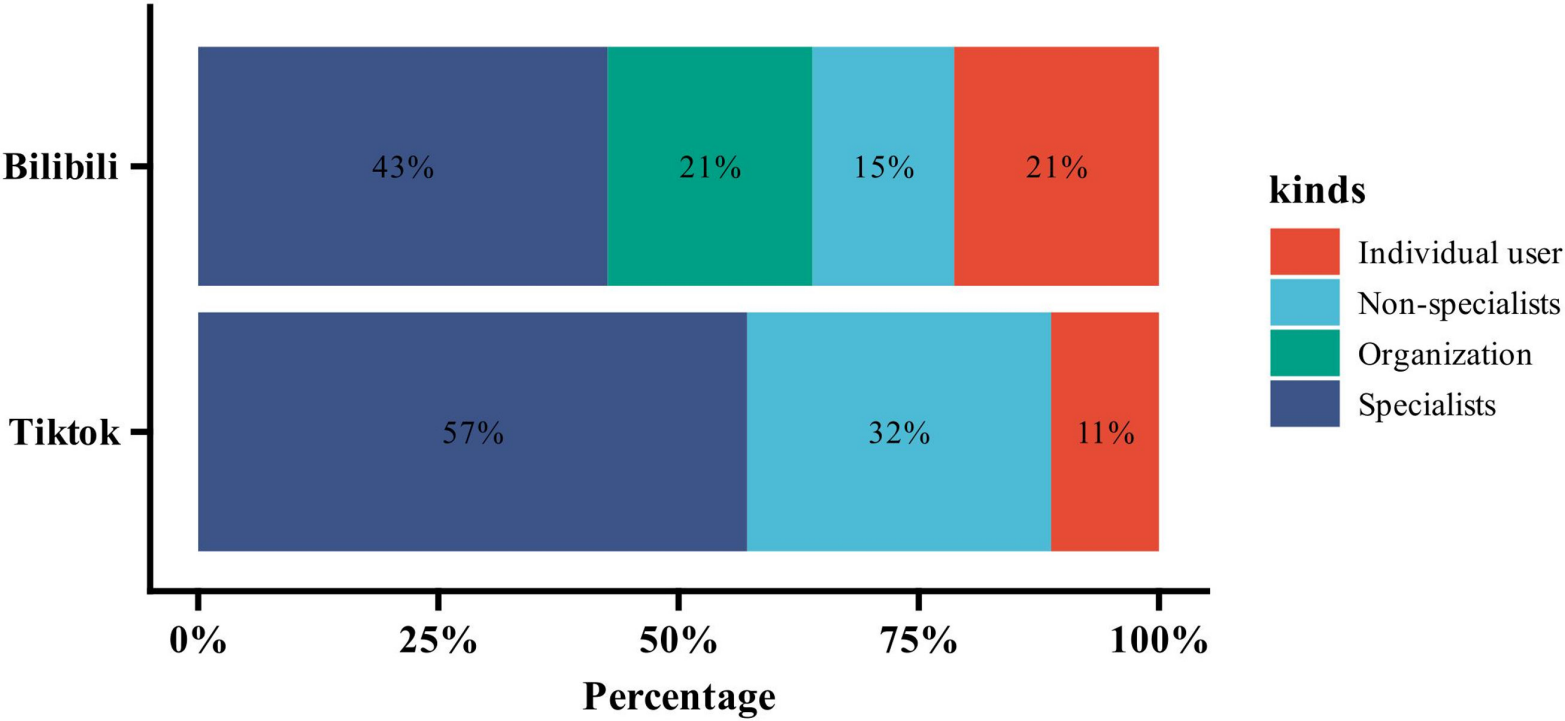
Proportion of different uploaders of lupus nephritis-related videos on Bilibili and TikTok.

The proportion of content creators varies across platforms. Figure 2 shows that professional medical practitioners constitute the largest proportion on Bilibili (43%) and TikTok (57%). The proportion of non-professional medical practitioners on Bilibili is the smallest (15%), while individual users account for the lowest proportion on TikTok (11%). Nonprofessional medical practitioners make up 32% on TikTok, and institutions account for 21% on Bilibili. There are no institutional publishers on TikTok. Among all individuals with medical knowledge (including professional and nonprofessional medical practitioners), Bilibili accounts for 79%, and TikTok accounts for 89%.

### 3.3. Interactivity and video quality assessment of different platforms

There are significant differences in video duration and audience interaction metrics between the 2 platforms (Table 1). TikTok videos are notably shorter (median: 82.5 seconds, IQR: 60.0–134.8), whereas Bilibili videos are longer (median: 486.0 seconds, IQR: 66.0–1886.0). However, TikTok videos perform better in audience interaction metrics, including higher counts of likes, comments, collections, and shares, indicating that they are more engaging and interactive. What is more, although both platforms have the same median GQS score of 2.00, the distribution of scores differs significantly between Bilibili (IQR: 2.00–3.00) and TikTok (IQR: 2.00–2.00) (*P* <.05) (Table 1). We find that when the variables are not adjusted, compared with the TikTok platform, the Bilibili platform is associated with a higher GQS score. The odds ratio value and 95% confidence interval are 2.11 (1.15–3.90). After adjusting for covariates such as video length, likes, collections, comments, shares, and uploader type, the odds ratio value and 95% confidence interval are 1.40 (0.43–4.64) (Table 2). In addition, there is no significant difference in the mDISCERN scores between the 2 platforms (*P* >.05), indicating that the levels of information accuracy, balance, and understandability are equivalent.

**Table 1:** Summary of length, engagement, and quality of lupus nephritis-related short videos on Bilibili and TikTok.

Variables	Bilibili (n = 61)	TikTok (n = 98)	*P*
Video length, M (Q_1,_ Q_3_)	486.00 (66.00, 1886.00)	82.50 (60.00, 134.75)	<.05
Likes, M (Q_1_, Q_3_)	23.00 (3.00, 120.00)	190.50 (103.00, 488.50)	<.05
Collections, M (Q_1_, Q_3_)	58.00 (5.00, 198.00)	65.50 (24.25, 171.50)	.23
Comments, M (Q_1_, Q_3_)	1.00 (0.00, 9.00)	22.00 (7.25, 85.75)	<.05
Shares, M (Q_1_, Q_3_)	14.00 (1.00, 43.00)	42.50 (14.00, 126.75)	<.05
GQS, M (Q_1_, Q_3_)	2.00 (2.00, 3.00)	2.00 (2.00, 2.00)	.05
mDISCERN, M (Q_1_, Q_3_)	2.00 (2.00, 3.00)	2.00 (1.25, 3.00)	1.00

GQS = Global Quality Score, mDISCERN = modified DISCERN.

**Table 2:** Logistic regression analysis of the quality of lupus nephritis-related short videos on Bilibili and TikTok.

	Reference	OR (95%CI)	*P*
GQS			
Model 1	TikTok	2.11 (1.15–3.90)	<.05
Model 2	TikTok	1.40 (0.43–4.64)	.66
mDISCERN			
Model 1	TikTok	1.00 (0.56–1.79)	1.00
Model 2	TikTok	0.78 (0.28–2.16)	.63

Model 1: no covariates were adjusted. Model 2: adjusted for covariates such as video length, likes, collections, comments, shares, and uploader type.

CI = confidence interval, GQS = Global Quality Score, mDISCERN = modified DISCERN, OR = odds ratio.

### 3.4. Interactivity and video quality assessment of different uploaders

**Figure 3.**
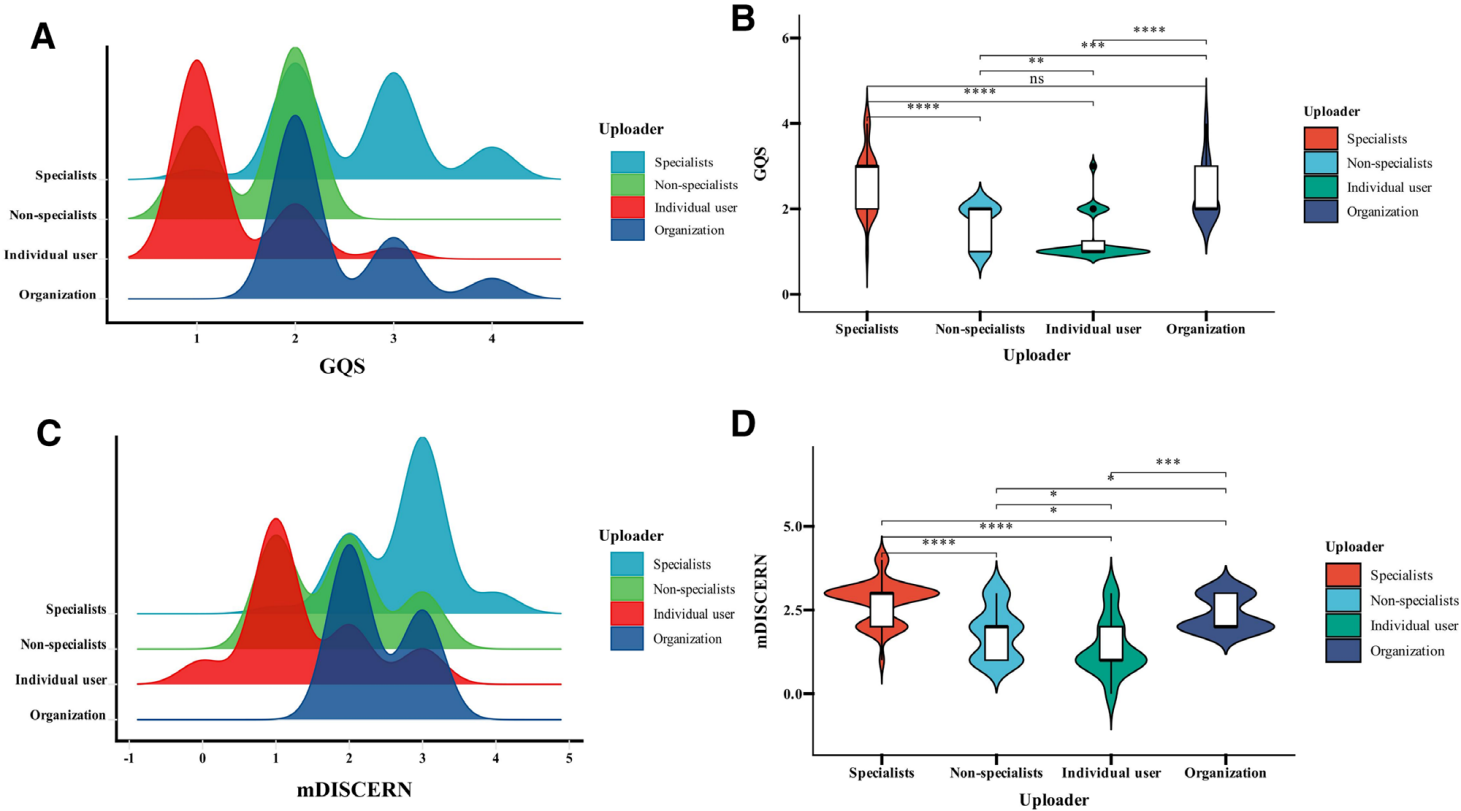
GQS and mDISCERN scores for short videos on lupus nephritis by different uploaders. (A) GQS score distribution by video uploader groups, (B) GQS score ratings by video uploader groups, (C) mDISCERN score distribution by video uploader groups, (D) mDISCERN score ratings by video uploader groups. GQS = Global Quality Score, mDISCERN = modified DISCERN.

When grouped by uploader type, the videos uploaded by institutions have the longest duration (1616.00 seconds) and the highest number of collections, but the lowest numbers of likes, comments, and shares. Individual users and nonspecialist clinicians are more interactive: individual users generate the most comments, and nonspecialist clinicians achieve the highest number of shares (Table 3). In addition, the content generated by experts obtains the highest GQS (median: 3.00, IQR: 2.00–3.00) and mDISCERN (median: 3.00, IQR: 2.00–3.00) scores. Individual users have the lowest GQS and mDISCERN scores, at 1.00 (1.00–1.25) and 1.00 (1.00–2.00), respectively. This shows that there is a positive correlation between professional background and content credibility (Table 3, Fig. 3).

**Table 3:** Summary of video length, engagement metrics, and quality for lupus nephritis-related content across different uploaders.

Variables	Nonspecialists (n = 40)	Individual user (n = 24)	Organization (n = 13)	Specialists (n = 82)	*P*
Video length, M (Q_1_, Q_3_)	73.00 (53.00, 114.00)	118.00 (57.75, 248.50)	1616.00 (50.00, 2297.00)	119.50 (65.25, 248.00)	.01
Likes, M (Q_1_–Q_3_)	186.50 (94.00, 346.50)	440.50 (29.00, 3604.75)	42.00 (2.00, 127.00)	103.50 (28.00, 259.00)	.02
Collections, M (Q_1_, Q_3_)	79.00 (28.50, 229.00)	61.50 (6.75, 639.50)	95.00 (7.00, 198.00)	49.00 (18.75, 103.00)	.54
Comments, M (Q_1_, Q_3_)	12.50 (5.75, 31.75)	135.00 (17.00, 483.50)	1.00 (0.00, 4.00)	9.00 (1.00, 33.00)	<.05
Shares, M (Q_1_, Q_3_)	39.50 (13.50, 132.50)	31.00 (2.75, 686.25)	19.00 (4.00, 53.00)	19.50 (6.00, 67.00)	.21
GQS, M (Q_1_, Q_3_)	2.00 (1.00, 2.00)	1.00 (1.00, 1.25)	2.00 (2.00, 3.00)	3.00 (2.00, 3.00)	<.05
mDISCERN, M (Q_1_, Q_3_)	2.00 (1.00, 2.00)	1.00 (1.00, 2.00)	2.00 (2.00, 3.00)	3.00 (2.00, 3.00)	<.05

GQS = Global Quality Score, mDISCERN = modified DISCERN.

### 3.5. Content analysis

**Figure 4.**
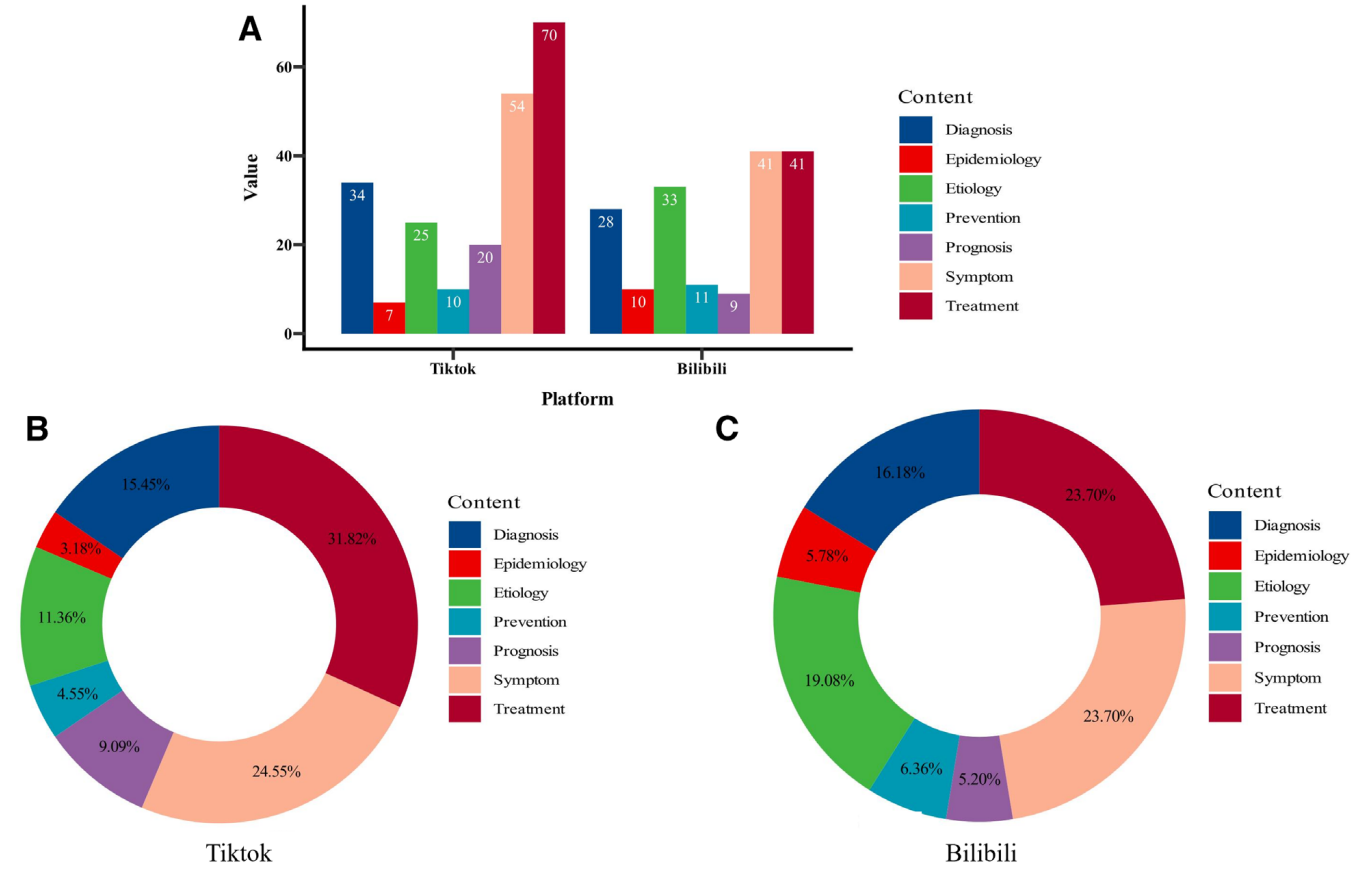
Proportion of different content types in Bilibili and TikTok short videos on lupus nephritis. (A) Video content distribution across the two platforms, (B) Percentage distribution of video content types on TikTok, (C) Percentage distribution of video content types on Bilibili.

Treatment-related content dominates TikTok, accounting for 31.82%, followed by symptoms (24.55%), diagnosis (15.45%), etiology (11.36%), prognosis (9.09%), prevention (4.55%), and epidemiology (3.18%). On Bilibili, treatment and symptoms each account for 23.70%, followed by etiology (19.08%), diagnosis (16.18%), prevention (6.36%), prognosis (5.20%), and epidemiology (5.78%; Fig. 4).

### 3.6. Correlation analysis

**Figure 5.**
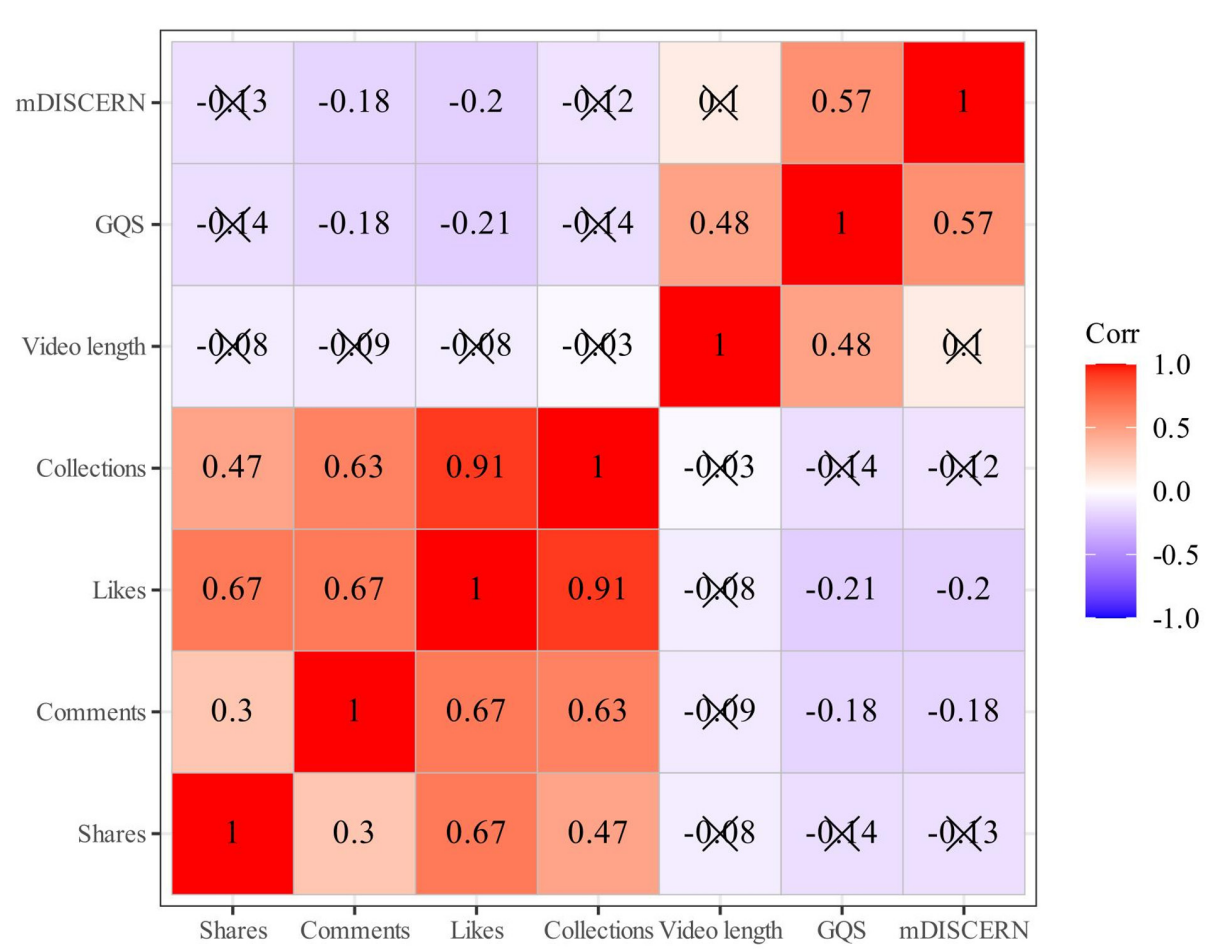
Correlation analysis chart for lupus nephritis-related short videos.

There is a positive correlation among video interaction metrics (shares, likes, collections, and comments). These interaction metrics are not related to video duration. Comments and likes are negatively correlated with video quality (GQS and mDISCERN). Positive correlations are observed between different video quality scores, as well as between video duration and GQS scores (Fig. 5).

## 4. Discussion

LN is an important cause of ESRD, and there are significant differences in the treatment methods and prognosis among different pathological subtypes. Therefore, early diagnosis and intervention are crucial for patients with LN.^[8,21]^ At present, more and more patients obtain disease-related information from short-video platforms, but the quality of such content is highly uneven.^[22–24]^ For the first time, this study systematically evaluated the quality and reliability of LN-related short videos on TikTok and Bilibili. The results show that Bilibili excels in longer videos dominated by medical professionals and provides higher information reliability, while TikTok achieves better interaction through concise content but has a lower quality score. The uploader’s professional background is positively correlated with content credibility, while high interactivity is negatively correlated with quality scores. In addition, most short videos focus on the symptoms and treatment of diseases, with limited coverage of epidemiology, prevention, and prognosis.

Our findings show that Bilibili’s GQS score is higher than TikTok’s, which is consistent with the results for prostate cancer and knee osteoarthritis.^[16,17]^ However, after adjusting for covariates, there is no significant difference in GQS scores between the 2 platforms. This suggests that Bilibili’s GQS score advantage is mainly influenced by covariates such as video duration, likes, and uploader type. Similarly, no significant difference is found in mDISCERN scores between the platforms, aligning with findings on premature ovarian insufficiency.^[15]^ TikTok mainly presents short-form content, which is observed to be associated with higher interactivity. The content focuses on fragmented information such as treatment and symptoms. In contrast, Bilibili’s longer video format is associated with more comprehensive content structures, including etiological analysis and prognostic discussion.

Specialist clinicians dominate the production of high-quality content. Although their video interaction metrics are moderate, they ensure accuracy, balance, and evidence-based support. In contrast, individual users produce the lowest-quality content but receive a high number of comments through emotional appeals. Nonspecialist clinicians and institutions show contradictory characteristics: the former post most frequently, but content quality is uneven; the latter provide detailed content but suffer from low interaction due to rigid formats. These findings suggest that higher medical professionalism is associated with improved video quality, which is consistent with the research conclusions on knee osteoarthritis.^[16]^ Consequently, users should prioritize viewing videos released by qualified medical practitioners on the platform. A potential future direction worth exploring could be the implementation of a “creator certification system” to highlight specialist content, which might help patients identify more reliable sources, although this suggestion requires further empirical testing.

Our research shows that treatment and symptoms are the most frequently discussed topics on TikTok and Bilibili, while prevention (Bilibili: 6.36%) and epidemiology (TikTok: 3.18%) have the lowest coverage. This bias may strengthen patients’ treatment-centered cognition and neglect long-term management and prevention of LN, thus increasing the risk of self-discontinuationof medication or inappropriate dietary adjustments. For example, only 11.36% of TikTok content involves the causes of the disease, which may lead patients to underestimate the role of environmental factors such as light exposure in disease onset. In addition, renal biopsy is still the basis of LN assessment and management, which can guide clinicians to choose appropriate treatment strategies.^[5]^ This is contrary to the clinical reality that LN requires meticulous management. Incorrect decision-making may accelerate the deterioration of renal function or induce infections.^[25–27]^ Medical institutions should give priority to establishing a presence on long-form video platforms like Bilibili and producing structured content (e.g., cause-treatment-prognosis).

Correlation analysis reveals a key paradox: audience interaction metrics (likes, comments, and shares) are positively correlated with each other, which is consistent with previous findings on the interdependence of user participation indicators on short-video platforms.^[12]^ However, these metrics are negatively correlated with GQS/mDISCERN scores (especially comments, which show a negative correlation with content quality), a pattern similar to that observed in studies of conditions such as gallstones.^[14,28]^ At the same time, video length is positively correlated with GQS (longer videos are considered more credible) but shows no significant correlation with interaction metrics. This shows that high interaction does not necessarily reflect content value; emotional or controversial content is easy to generate comments but may lack scientific rigor, while expert-produced videos, although methodologically sound, may have limited reach due to technical terminology. This finding challenges the hypothesis that “interaction is equal to influence” and advocates for the introduction of a “quality-dissemination trade-off metric” in health communication. In fact, if platforms simply optimize interaction algorithms, low-quality information may be more likely to circulate and be preferentially recommended, potentially making it easier for patients to access such content. By integrating tools like GQS/mDISCERN into platform audit standards, misleading LN can be filtered out at the source.

This study has limitations. First, this study employs a cross-sectional design, which precludes causal inference and permits only the identification of associations observed concurrently. Second, the sample covers only 2 major platforms (159 videos). In addition, excluding videos from the past week may ignore emerging trends and fail to account for cultural factors, such as content related to traditional Chinese medicine. Future research should expand the scope of platforms; cover Chinese and English platforms, including international channels such as YouTube; explore artificial intelligence-assisted real-time quality monitoring systems; and conduct longitudinal tracking of content updates to assess their impact on health outcomes.

In summary, this study reveals a complementary pattern between the 2 platforms in LN health communication: Bilibili videos are characterized by higher reliability but lower reach in terms of user engagement, whereas TikTok videos show broader reach but relatively lower depth and quality scores. Specialist clinicians are the cornerstone of quality, but a multi-platform strategy needs to be adopted to balance rigor and communication. Future efforts may improve the digital health ecosystem through standardized content evaluation mechanisms and increased participation of professional medical creators.

## Acknowledgments

The authors gratefully acknowledge the support of the Hunan Province’s Natural Science Foundation (Grant No. 2024JJ6564) and Scientific Research Launch Project for new employees of the Second Xiangya Hospital of Central South University (Grant No. QH20230228).

## Author contributions

**Conceptualization:** Xuemin Yin.

**Software:** Xi Fang.

**Writing – original draft:** Xuemin Yin.

**Writing – review & editing:** Yinjun Liao.




