# Supplementary figures and images for: Quality assessment of lupus nephritis health information on China’s mainstream short-form video platforms: A cross-sectional study
Source: Medicine (Baltimore). 2026 Jul 24;105(30):e49924. doi: 10.1097/MD.0000000000049924 (PMC13406252; doi:10.1097/MD.0000000000049924)

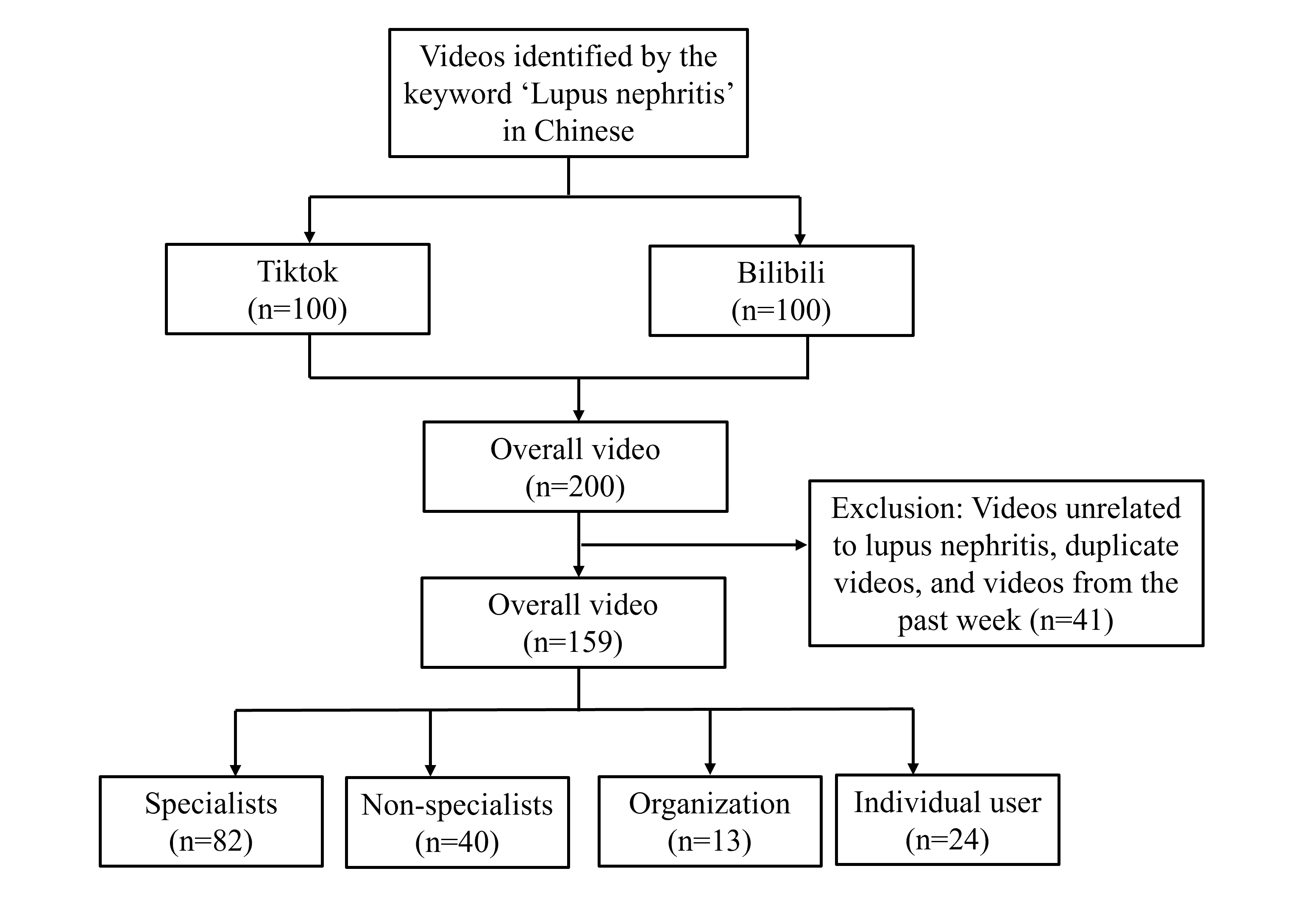

Supplement: Supplementary file 1 [file medi-105-e49924-s001.tiff]
