# Supplementary material for: Quality assessment of lupus nephritis health information on China’s mainstream short-form video platforms: A cross-sectional study
Source: Medicine (Baltimore). 2026 Jul 24;105(30):e49924. doi: 10.1097/MD.0000000000049924 (PMC13406252; doi:10.1097/MD.0000000000049924)
Supplement: Supplementary file 2 [file medi-105-e49924-s002.doc]

| Characteristic | Total (n = 159) |  |
| --- | --- | --- |
|  |  |  |
| Platform, n (%) |  |  |
|  | Tiktok 98 (61.64) |  |
|  | Bilibili 61 (38.36) |  |
| Video source, n (%) |  |  |
|  | Specialists 82 (51.57) |  |
|  | Non-specialists 40 (25.16) |  |
|  | Organization 13 (8.18) |  |
|  | Individual user 24 (15.09) |  |
| Likes, M (Q₁, Q₃) | 127.00 (35.00, 339.50) |  |
| Collections, M (Q₁, Q₃) | 61.00 (17.50, 181.50) |  |
| Comments, M (Q₁, Q₃) | 11.00 (2.00, 72.00) |  |
| Shares, M (Q₁, Q₃) | 26.00 (6.50, 88.00) |  |
| Video length, M (Q₁, Q₃) | 140.00 (83.00, 302.00) |  |
| mDISCERN, M (Q₁, Q₃) | 2.00 (2.00, 3.00) |  |
| GQS, M (Q₁, Q₃) | 2.00 (2.00, 3.00) |  |
| Epidemiology, n (%) | 17 (4.33) |  |
| Etiology, n (%) | 58 (14.76) |  |
| Symptoms, n (%) | 95 (24.17) |  |
| Diagnosis, n (%) | 62 (15.78) |  |
| Treatment, n (%) | 111 (28.24) |  |
| Prevention, n (%) | 21 (5.34) |  |
| Prognosis, n (%) | 29 (7.38) |  |

Supplementary Table 1. Summary of information in lupus nephritis-related Videos
